# Expectation Cues and False Percepts Generate Stimulus-Specific Activity in Distinct Layers of the Early Visual Cortex

**DOI:** 10.1523/JNEUROSCI.0998-23.2023

**Published:** 2023-11-22

**Authors:** Joost Haarsma, Narin Deveci, Nadege Corbin, Martina F. Callaghan, Peter Kok

**Affiliations:** ^1^Wellcome Centre for Human Neuroimaging, University College London Queen Square Institute of Neurology, University College London, London WC1N 3AR, United Kingdom; ^2^Centre de Résonance Magnétique des Systèmes Biologiques, Unité Mixte de Recherche 5536, Centre National de la Recherche Scientifique, Université de Bordeaux, 33076 Bordeaux, France

**Keywords:** false percepts, laminar fMRI, predictive coding

## Abstract

Perception has been proposed to result from the integration of feedforward sensory signals with internally generated feedback signals. Feedback signals are believed to play an important role in driving false percepts, that is, seeing things that are not actually there. Feedforward and feedback influences on perception can be studied using layer-specific fMRI, which we used here to interrogate neural activity underlying high-confidence false percepts while healthy human participants (*N* = 25, male and female) performed a perceptual orientation discrimination task. Auditory cues implicitly signaled the most likely upcoming orientation (referred to here as expectations). These expectations induced orientation-specific templates in the deep and superficial layers of V2, without affecting perception. In contrast, the orientation of falsely perceived stimuli with high confidence was reflected in the middle input layers of V2, suggesting a feedforward signal contributing to false percepts. The prevalence of high-confidence false percepts was related to everyday hallucination severity in a separate online sample (*N* = 100), suggesting a possible link with abnormal perceptual experiences. These results reveal a potential feedforward mechanism underlying false percepts, reflected by spontaneous stimulus-like activity in the input layers of the visual cortex, independent of top-down signals reflecting cued orientations.

**SIGNIFICANCE STATEMENT** False percepts have been suggested to arise through excessive feedback signals. However, feedforward contributions to false percepts have remained largely understudied. Laminar fMRI has been shown to be useful in distinguishing feedforward from feedback activity as it allows the imaging of different cortical layers. In the present study we demonstrate that although cued orientations are encoded in the feedback layers of the visual cortex, the content of the false percepts are encoded in the feedforward layers and did not rely on these cued orientations. This shows that false percepts can in principle emerge from random feedforward signals in the visual cortex, with possible implications for disorders hallmarked by hallucinations like schizophrenia and Parkinson's disease.

## Introduction

Our perception is not always veridical and can become distorted or biased in a myriad of ways, leading to false perceptual inferences. However, the neural mechanisms underlying such false inferences remain hotly debated, with some theories highlighting the contribution of top-down influences such as perceptual expectations, whereas others have pointed out a possible role for feedforward mechanisms. Elucidating the various ways perception can go awry is crucial as it sheds new light on false perceptual inferences as seen in psychiatric disorders like psychosis and neurologic disorders like Parkinson's disease (Weil et al., 2016).

In recent years, there has been growing interest in how our expectations affect the way we perceive the world, leading to potential false inferences (Corlett et al., 2019; Powers et al., 2016; Reichert et al., 2013; Sterzer et al., 2018). Predictive coding has emerged as a popular framework for understanding these false inferences (Sterzer et al., 2018). According to this theory, the brain continuously generates expectations about the world, which are sent to sensory cortices through neural feedback connections to be combined with sensory inputs in an effort to form an accurate representation of the world (Bastos et al., 2012; Friston, 2009). Rather than using a colloquial definition of the term expectation, that is, explicitly held beliefs about the future, more typically these frameworks (as well as the present article) uses a statistical definition of expectation, that is, predicted observations given a model of the world. In this sense it encompasses various ways in which prior knowledge shape the way the world is perceived, be it implicit or explicit, low level or high level (Aitken et al., 2020a; Chalk et al., 2010; de Lange et al., 2018; Kok et al., 2013). In these frameworks, when these expectations are overly strong, they can override sensory input and lead to false perceptions (Corlett et al., 2019; Haarsma et al., 2022; Powers et al., 2016; Reichert et al., 2013; Sterzer et al., 2018). Numerous studies have shown that expectations have a significant role in shaping our sensory experiences (de Lange et al., 2018). Additionally, research suggests that individuals experiencing hallucinations exhibit a stronger influence of expectations on perception (Cassidy et al., 2018; Haarsma et al., 2020; Kafadar et al., 2020; Powers et al., 2017; Schmack et al., 2013, 2015, 2021; Stuke et al., 2021; Teufel et al., 2015).

However, it is unclear if all false perceptions are because of feedback influences or if they can also arise from feedforward mechanisms. This question is important for understanding different pathways to hallucinations. Studies have shown that in some neurologic disorders, like Charles Bonnet syndrome, hallucinations can result from changes in feedforward sensory signals (Burke, 2002; Hahamy et al., 2021). Spontaneous activity in the early visual cortex can generate hallucinatory perceptions in this syndrome, possibly following the visual cortex becoming partially deafferented (Burke, 2002; Desai et al., 1999; Painter et al., 2018; Reichert et al., 2013). Further, studies in psychosis suggest an increased reliance on sensory evidence, with a particular relation to delusions (Schmack et al., 2013). Spontaneous activity may contribute to false perceptions in healthy individuals as well, but it is uncertain if these arise from feedforward or feedback signals (Boly et al., 2007; Busch et al., 2009; Pajani et al., 2015; Podvalny et al., 2019; Wyart and Tallon-Baudry, 2009).

Feedforward signals preferentially terminate in the middle layers of the visual cortex, whereas feedback signals terminate in the deep and superficial (i.e., agranular) layers (Felleman and Van Essen, 1991; Harris and Mrsic-Flogel, 2013). Therefore, measuring activity in the different layers using layer-specific fMRI enables testing theories about the contribution of feedforward and feedback signals to perceptual phenomena (Haarsma et al., 2022; Jia et al., 2020; Kok et al., 2016; Lawrence et al., 2019b; Muckli et al., 2015; Ng et al., 2021; Self et al., 2019; Stephan et al., 2019). Here, we used layer-specific fMRI to investigate the cortical layers involved in representing false perceptions of oriented gratings. If such false percepts are driven by feedback activity, they should be reflected by orientation-specific activity in the agranular layers of the visual cortex, whereas if false percepts are driven by feedforward activity they should be reflected by activity in the middle layers of the visual cortex. To preview our results, although the most likely orientations cued by an auditory stimulus (which we dubbed expectations) were indeed signaled in the deep and superficial layers, false percepts were reflected in the middle layers of the early visual cortex, suggesting that false percepts can arise from endogenous fluctuations in feedforward signals.

## Materials and Methods

### Ethics statement

This study was approved by the University College London Research Ethics Committee (R13061/RE002 for the imaging study, R6649/RE004 for the online study) and was conducted according to the principles of the Declaration of Helsinki. All participants gave written informed consent before participation and received monetary compensation (£7.50 an hour for behavioral tasks, £10 an hour for MRI).

### Participants

Twenty-eight healthy human volunteers with normal or corrected-to-normal vision participated in the 7T fMRI experiment. Three participants were excluded because of our strict head motion criteria of no more than 10 movements larger than 1.0 mm in any direction between successive functional volumes. For the remaining participants, the maximum change in head position in any direction over the course of the fMRI runs was within 4 mm (0.66 ± 0.54 mm, mean ± SD over participants) of the mean head position (to which the anatomic boundaries were registered). The final sample consisted of 25 participants (22 female, age 25 ± 4 years, mean ± SD). One hundred participants participated in the online study. Participants were recruited through Prolific (https://www.prolific.co) and were paid £7.50 for their participation.

### Questionnaires

For the online study, questionnaire data were collected for the Peter et al. Delusions Inventory (PID; Peters et al., 2004), as well as the Cardiff Anomalous Perceptions Scale (CAPS; Bell et al., 2006). Total scores were calculated for the PID and CAPS by adding their respective subscales. These were then correlated with the behavioral measures for the online study.

### Stimuli

Grayscale luminance-defined sinusoidal Gabor grating stimuli were generated using MATLAB (MathWorks; RRID:SCR_001622) and the Psychophysics Toolbox (Brainard and Vision, 1997). During the behavioral session for the fMRI study, the stimuli were presented on a PC (1920 × 1200 screen resolution, 60 Hz refresh rate). In the fMRI scanning session, stimuli were projected onto a rear projection screen using an Epson EB-L1100U Laser Projector (1920 × 1200 screen resolution, 60 Hz refresh rate) and viewed via a mirror (viewing distance 91 cm). On grating-present trials (50%), auditory cues were followed by a grating after a 750 ms delay (0.5 cycles per degree spatial frequency, 33 ms duration), displayed in an annulus (outer diameter, 10° of visual angle; inner diameter, 1°; contrast decreasing linearly to 0 over 0.7° at the inner and outer edges), surrounding a fixation bull's eye (0.7° diameter). These stimuli were combined with one of four noise patches, which resulted in a 4% contrast grating embedded in 20% contrast noise during the fMRI session. On grating-absent trials, one of the four noise patches was presented on its own. Noise patches were created through smoothing pixel-by-pixel Gaussian noise with a Gaussian smoothing filter, ensuring that the spatial frequency of the noise patches matched that of the gratings. This was done to ensure that the noise patches and gratings had similar low-level properties, increasing the likelihood of reporting false percepts. To avoid including noise patches that contained grating-like orientation signals by chance, the noise patches were processed through a bank of Gabor filters with varying preferred orientations. Only noise patches with low (2%) signal energy for all orientations were selected to be included in the present experiment. The resulting four noise patches were used for all trials throughout the experiment, in a counterbalanced manner, ensuring that reported false percepts could only be triggered by internal mechanisms (Pajani et al., 2015; Wyart et al., 2012). During the practice session on the first day, the contrast of the gratings was initially high (80%), gradually decreasing to 4% toward the end of the practice. The central fixation bull's-eye was present throughout the trial, as well as during the intertrial interval (ITI; jittered exponentially between 2150 and 5,150 ms). In the online study, multiple grating contrast levels were presented, titrated to each individual (see below, Experimental procedure).

### Experimental procedure

On the first day of testing for the laminar fMRI study, participants underwent a behavioral practice session. The practice consisted of an instruction phase with 7 blocks of 16 trials in which the task was made progressively more difficult while verbal and written instructions were provided. During these practice runs, the auditory cues predicted the orientation of the grating stimulus with 100% validity (45° or 135°; no grating-absent trials). After the completion of the instructions, the participants completed 4 runs of 128 trials each, separated into 2 blocks of 64 trials each. In the first two runs the expectation cues were 100% valid to ensure participants learned the association, whereas in the final two runs the cues were 75% valid (i.e., the grating had an unexpected orientation on 25% of trials) to test whether participants might have adopted a response bias. Grating contrast decreased over the four runs; specifically, the contrast levels were 7.5, 6, 5, and 4%, whereas the contrast of the noise patches remained constant at 20%. No grating-absent trials were presented on day 1. On the second day, participants performed the same task in the MR scanner. As on the first day, four runs were completed, but now the grating contrast was fixed at 4% on grating-present trials, and on 50% of the trials the gratings were omitted and only noise patches were presented, resulting in grating-absent trials. On grating-present trials the cues always predicted the orientation of the grating with 100% validity. On grating-absent trials the cue was by definition invalid as no grating orientation was presented. Each run lasted ∼12.5 min, totaling ∼50 min.

Trials consisted of an auditory expectation cue, followed by a grating stimulus embedded in noise on 50% of trials (750 ms stimulus onset asynchrony between cue and grating). The auditory cue (a high or low tone) predicted the orientation of the grating stimulus (45° or 135°). On grating-present trials, a grating with the orientation predicted by the auditory cue was presented embedded in noise, whereas on grating-absent trials only a noise patch was presented. The stimulus was presented for 33 ms in the fMRI study. After the stimulus disappeared, the orientation response prompt appeared, consisting of a left- and right-pointing arrow on either side of the fixation dot (location was counterbalanced). Participants were required to select the arrow corresponding to their answer (left arrow for anticlockwise or 135°, right arrow for clockwise or 45°; 1 s response window) through a button press with their right hand. Subsequently the letters CONF? appeared on the screen probing participants to indicate their confidence that they had seen a grating (1 = I did not see a grating, 2 = I may have seen a grating, 3 = I probably saw a grating, 4 = I am sure I saw a grating), using one of four buttons with their left hand (1.25 s response window). Participants indicated their response using an MR-compatible button box in the MRI scanner and a keyboard during training.

After the main experiment, participants performed a functional localizer task inside the scanner. This consisted of flickering gratings (2 Hz), presented at 100% contrast in blocks of ∼14.3 s (four TRs). Each block contained gratings with a fixed orientation (45° or 135°). The two orientations were presented in a pseudorandom order followed by an ∼14.3 s blank screen containing only a fixation bull's-eye. Participants were tasked with responding when the black fixation dot briefly dimmed to ensure central fixation. All participants were presented with 16 localizer blocks, which totaled ∼15 min.

The online study was created and hosted using Gorilla Experiment Builder (https://gorilla.sc; Anwyl-Irvine et al., 2020), and participants were recruited through Prolific as stated above. Before the start of the instruction blocks participants were asked to keep a 50 cm distance from the screen. They were required to adjust the size of a rectangle on their screen to match a bank card to ensure that the visual angle was equal across participants. Subsequently, they were asked to adjust the volume of their headphones to a high but not unpleasant volume. The instruction phase of the online study was the same as the instruction phase of the fMRI study. The timings for the trials were identical as well, except that the stimuli appeared on the screen for 50 ms instead of 33 ms because of software constraints. After completing the seven instruction blocks, participants were required to complete four blocks with different grating contrast levels (7.5, 6, 5, and 4%, in that order). The lowest grating contrast for which participants were able to perform the orientation task with at least 75% accuracy served as the base contrast value for the main experiment. During the main experiment, participants were required to complete four blocks of 128 trials. Unlike in the fMRI experiment, different grating contrast levels were presented, and expectation cues were sometimes invalid (6.7% of grating present trials). Specifically, of the 128 trials on each block, on 96 trials (75%) a grating was present and on 32 (25%) only a noise patch was presented. Of the 96 grating-present trials, one-third (32) was presented at the base contrast, one-third at base − 1% contrast, and the other third at base + 1% contrast. Of these grating-present trials, 90 (93.3%) were valid, and 6 (6.7%) were invalid.

### fMRI data acquisition

MRI data were acquired on a Siemens MAGNETOM Terra 7T MRI system (Siemens Healthcare) with an eight-channel head coil for localized transmission, operating in a quadrature-like (TrueForm) mode, with a 32-channel head coil insert for reception (Nova Medical) at the Wellcome Center for Human Neuroimaging, University College London. Functional images were acquired using a T2*-weighted 3D gradient-echo EPI sequence [volume acquisition time of 3,552 ms, TR = 74 ms, TE = 26.95 ms; voxel size, 0.8 × 0.8 × 0.8 mm^3^; 15° flip angle; field of view, 192 × 192 × 38.4 mm^3^; Generalized Autocalibrating Partial Parallel Acquisition (GRAPPA) acceleration factor 4; and partial Fourier 6/8 in the phase-encoded direction of the EPI readout, binomial (1331) water-selective excitation]. Anatomical images were acquired using a magnetization-prepared two rapid acquisition gradient echo (MP2RAGE) sequence (TR = 5,000 ms, TE = 2.54 ms, TI = 900 ms and 2,750 ms; voxel size, 0.65 × 0.65 × 0.65 mm; 5° and 3° flip angles; field of view, 208 × 208 × 156 mm^3^; in-plane GRAPPA acceleration factor 3).

### Preprocessing of fMRI data

The first two volumes of each run were discarded. Before registration the functional volumes were cropped to cover only the occipital lobe to reduce the influence of severe distortions in the frontal lobe. The cropped functional volumes were spatially realigned within scanner runs, and subsequently between runs, to correct for head movement using Statistical Parametric Mapping (SPM)12 software (https://www.fil.ion.ucl.ac.uk/spm/).

### Segmentation and coregistration of cortical surfaces

The methods for segmenting and coregistering cortical surfaces are identical to several previously published studies (Aitken et al., 2020b; Kok et al., 2016; Lawrence et al., 2018, 2019a) and are reiterated here. FreeSurfer (http://surfer.nmr.mgh.harvard.edu/) was used to detect the gray matter (GM) and white matter (WM) boundaries and CSF based on a bias-corrected MP2RAGE image. The boundaries were checked for errors where the dura was mistakenly included in the pial surface. Subsequently the GM boundaries were registered to the mean functional image. Specifically, a conventional rigid-body registration was followed by a recursive boundary-based registration (RBR; van Mourik et al., 2019). RBR consisted of applying boundary-based registration (BBR) recursively to increasingly smaller partitions of the cortical mesh. An affine BBR was applied with seven degrees of freedom, rotation and translation along all three dimensions and scaling along the phase-encoding direction only. This scaling allows correction of distortions along the low bandwidth phase-encoded EPI direction of acquisition. In each iteration, the cortical mesh was split into two, and the optimal BBR transformations were found and applied to the respective parts. Subsequently, each part was split into two again and registered. The specificity increased at each stage and corrected for local mismatches between the structural and the functional volumes that are because of magnetic field inhomogeneity-related distortions. Six such iterations were performed. The splits were made along the cardinal axes of the volume, such that the number of vertices was equal for both parts. The plane for the second cut was orthogonal to the first, the third was orthogonal to the first two. The median displacement was taken after running the recursive algorithm six times in which different splitting orders where used, comprising all six permutations of *x*, *y*, and *z*.

### Definition of regions of interest

The definition of regions of interests (ROIs) was identical to that in a previously published study (Aitken et al., 2020b) and reiterated here. Primary visual cortex (V1) and secondary visual cortex (V2) surface labels were obtained through FreeSurfer, based on the segmentation of the MP2RAGE image (see Fig. 2*a*, left, gray voxels). These were subsequently projected to volume space, covering the full cortical depth plus a 50% extension into WM and CSF. Note that this interpolation from surface space to volume space led to some overlap between the V1 and V2 ROIs, precluding strong claims about effects being specific to V1 or V2. The V1 and V2 ROIs were subsequently constrained to the voxels that were responsive to the localizer gratings (see Fig. 2*a*, left, green voxels). Specifically, separate regressors were defined for the blocks of 45° and 135° gratings, respectively, and the mean of the resulting parameter estimates was contrasted against baseline to identify voxels that exhibited a significant response to the grating stimuli regardless of orientation (*t* > 2.3; V1, mean = 6208, SD = 1799 voxels; V2, mean = 9370, SD = 3256 over participants). Subsequently, the orientation preference of each voxel was estimated by contrasting the two orientation regressors from the localizer run. The 500 voxels that most strongly favored the 45° and 135° gratings during the localizer constituted the two orientation-specific ROIs within V1 and V2; see Fig. 2*a*, middle, purple and pink voxels). Finally, the time course of each voxel was normalized (*z*-scored) and multiplied by the absolute *t* value of the orientation contrast (45° vs 135°), to weight the data by the most robust orientation preference. Note that all reported effects in the *z*-scored data were also present without *z*-scoring. These ROI definitions were identical to those used in previous studies that successfully resolved orientation-specific BOLD signals with layer specificity (Aitken et al., 2020b; Lawrence et al., 2018, 2019a). The analysis approach was matched to these previous studies to facilitate comparisons between previous findings that involve orientation- and layer-specific fMRI signals.

### Definition of the cortical layers

GM was divided into three equivolume layers using the level set method described in detail previously (Kleinnijenhuis et al., 2015; van Mourik et al., 2019; Waehnert et al., 2014), following the principle that the layers of the cortex maintain their volume ratio throughout the curves of the gyri and sulci (Waehnert et al., 2014; see Fig. 2*a*, right). Briefly, the level set function is a signed distance function (SDF), where points on the same surface equal zero, values on one side of the surface are negative, and values on the other are positive. The level set function for the GM–CSF and GM–WM boundaries is calculated, and then intermediate surfaces can be defined by moving the surface to intermediate cortical depths. The equivolume model transforms a desired volume fraction into a distance fraction, taking the local curvature of the pial and WM surfaces at each voxel into account (van Mourik et al., 2019). Two intermediate surfaces between the WM and pial boundaries were calculated, yielding three GM layers (deep, middle, and superficial). In human early visual cortex, these three laminar compartments are expected to correspond roughly to layers I–III, layer IV, and layers V–VI, respectively (de Sousa et al., 2010). Based on these surfaces, four SDFs were calculated, containing for each functional voxel its distance to the boundaries between the five compartments (WM, CSF, and the three GM layers). This set of SDFs (or level set) allowed the calculation of the distribution of the volume of each voxel over the five compartments (van Mourik et al., 2019; see Fig. 2*b*). This layer volume distribution provided the basis for the laminar GLM discussed below.

### Extraction of layer-specific time courses

Because the fMRI data consisted of 0.80 mm isotropic voxels, individual voxels will naturally contain signals from multiple layers as well as WM and CSF (see Fig. 2*b*). Thus, if we were to simply interpolate the fMRI signal at different depths, there will be contamination from bordering layers. One way to address this so-called partial volume problem is to decompose the layers by means of a spatial GLM (Aitken et al., 2020b; Kok et al., 2016; Lawrence et al., 2019a; van Mourik et al., 2019). For every ROI (45, a laminar design matrix ***X*** represents the distribution of the 500 voxels over the different layers (*n* × *k*, where *n* = 500 voxels and *k* = 5 laminar compartments). Every row of ***X*** indicates the proportions of the layers covered by a particular voxel, and the columns represent the volume of the corresponding layer across voxels. This laminar design matrix can be used in a spatial GLM to separate the BOLD signal of the five different laminar compartments (three GM layers, WM, and CSF) through ordinary least-squares regression (van Mourik et al., 2019) as follows: Y=X⋅B +ε.

Here, ***Y*** is a vector of voxel values from an ROI in a specific functional volume, ***X*** is the laminar design matrix, and ***B*** is a vector of layer signals. For each ROI and each functional volume, the layer signal $\hat{B}$ was estimated by regressing ***Y*** against ***X***, yielding five depth-specific time courses per ROI.

To confirm that the method correctly identified GM, the raw signal in the EPI volumes for each of the three GM layers was quantified as well as WM and CSF. As expected, the signal intensity was higher in the three GM layers (deep, 239 ± 38; middle, 240 ± 46; superficial, 239 ± 40; mean ± SD over participants) than in WM (209 ± 30) and CSF (230 ± 51; *t*_(24)_ = 5.69, *p* = 7.4 × 10^−6^).

Crucially, the result of this spatial GLM is that we now have BOLD time courses for both 45°- and 135°-preferring voxels separate for superficial middle and deep layers of the early visual cortex. Now we can estimate our effects of interest for these time courses to explore which layers represent high-confidence false percepts and expectations.

#### Estimation of layer- and orientation-specific activity

A temporal GLM was used to estimate the effects of interest in each of the three GM layers. We separately modeled the two effects of interest, namely expecting and falsely perceiving specific orientations. For the expectations, we modeled the effect of expecting 45° or 135° orientations on grating-absent trials, resulting in two regressors. For the false percepts we modeled the effect of perceiving 45° or 135° orientations with high or low confidence on grating-absent trials, resulting in four regressors (see Fig. 2*d*,*e*). These regressors of interest were constructed by convolving stick functions representing the onsets of the trials with SPM12 canonical hemodynamic response function as well as their temporal derivative, resulting in beta values for each experimental effect. Furthermore, the head motion parameters, their derivatives, and the square of the derivatives were included as nuisance regressors. Subsequently, the data and the design matrix were high-pass filtered (cutoff = 128 s) to remove any low-frequency signal drifts.

To calculate orientation-specific BOLD responses, the layer-specific parameter estimates for each orientation in the noncorresponding ROI (e.g., a 45° grating/expectation in a 135°-preferring ROI) were subtracted from the parameter estimates in their corresponding ROI. By removing activity that is not specific to the perceived or expected orientation from activity in orientation congruent voxels, we get a measure of orientation-specific activity (e.g., a 45° grating/expectation in a 45°-preferring ROI; where B is for beta; Fig. 2*d*,*e*) as follows:

OrientationSpecificEffect=(45B−135B)45ROI + (135B−45B)135ROI.

Importantly, this equation results in activity specific for the expected or perceived percept (depending on the modeled effect). This procedure was followed for all the laminar analyses presented in this study (perceptual expectations, high-confidence false percepts, low-confidence false percepts). These estimated BOLD responses were subjected to a two-way repeated-measures ANOVA with factors perceptual condition (expectation, high-confidence false percept, low-confidence false percept), and cortical layer (deep, middle, superficial). The main effect of interest, namely, whether laminar BOLD profiles differed for perceptual expectations and hallucinated gratings, was tested by the interaction of perceptual condition and cortical layer. To follow up a significant effect with all three perceptual conditions included, further repeated-measures effects were performed to specifically test (1) the interaction between perceptual expectation versus high-confidence false percept and cortical layer to explore whether hallucinations were specifically different from perceptual expectations and (2) the interaction between high- and low-confidence false percepts and cortical layer to explore whether being confident in a false percept affects the laminar profile. Significant interactions were followed up with paired-sample *t* tests. Finally, orientation-specific effects in specific layers were tested against zero using one-sample *t* tests (one tailed). The rational for using one-tailed *t* tests here specifically is because orientation-specific activity is expected and is only interpretable with values above zero. To visualize the relevant across-subject variance for the within-subject ANOVA, error bars in all figures show within-subject SEM (Cousineau, 2005; Morey, 2008).

#### Behavioral analyses

For the online study, accuracy and confidence scores were compared across the different contrast levels using repeated-measures ANOVAs. Accuracy was also compared between the different confidence levels to test whether participants were more accurate at identifying grating orientation when they were more confident that they had seen a grating. The effect of the expectation cues was assessed by exploring whether participants tended to report orientations in line with the cue. Follow-up tests were performed to investigate whether the effects of the cues were mediated by awareness of their meaning. To understand what drives abnormal perceptual experiences, a logistic regression model was used to explore which factors predicted orientation responses on grating-present and grating-absent trials separately. Predictors for grating-present trials were current stimulus orientation, current stimulus contrast, orientation predicted by the cue, orientation response on the previous trial, and the interaction between present stimulus contrast and orientation (as a measure of sensory precision). For the grating-absent trials, the predictors included previous orientation response and orientation predicted by the cue (as there was no present stimulus orientation or contrast). Finally, we tested whether abnormal perceptual experiences as measured using the CAPS questionnaire were correlated with cue effects, confidence on grating-absent trials, and sensory precision, using Spearman's rank correlation.

Similarly, for the fMRI study, we probed the modulation of accuracy by confidence, the proportion of high-confidence false percepts on grating-absent trials, and the proportion of cue congruent responses. Participants' orientation responses were also explored with a logistic regression model, but without stimulus contrast as a predictor, as this was not varied for the purposes of the fMRI experiment.

## Results

### Participants experienced false percepts that were independent of perceptual expectation cues

**Figure 1.**
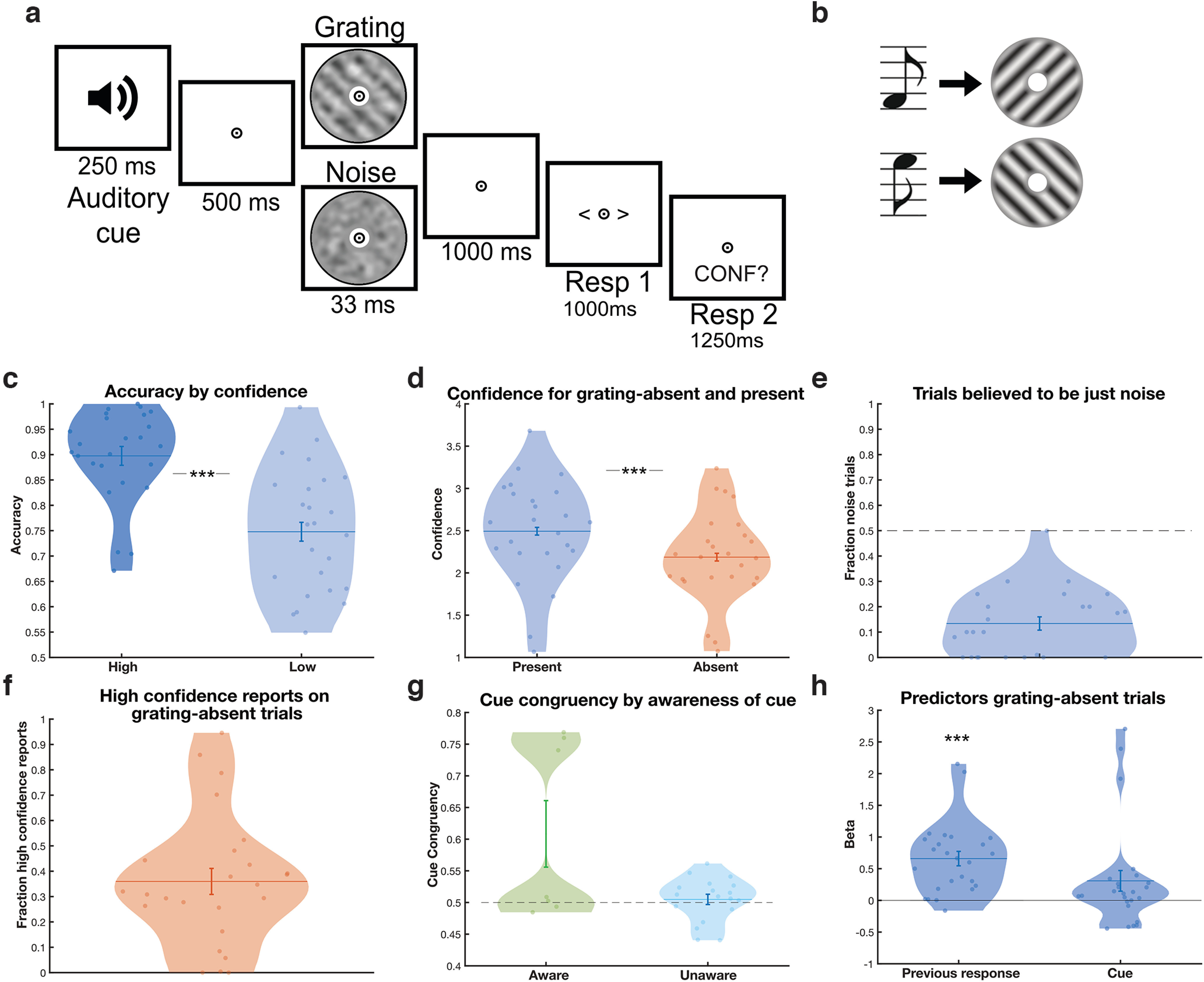
Experimental design and behavioral findings of a layer-specific fMRI study. ***a***, During the experiment, an auditory cue was followed by either a low-contrast grating embedded in noise (50% of trials) or a noise patch (50%). Participants indicated which orientation they saw and how confident they were that a grating was presented. ***b***, One sound predicted the appearance of a 45°, or clockwise, oriented grating, whereas the other predicted a 135°, or anticlockwise, oriented grating. Auditory cues were 100% valid on grating-present trials. ***c***, On grating-present trials, participants' orientation responses were more accurate when they indicated they were confident (dark blue) compared with not confident (light blue) they had seen a grating. ***d***, Participants were more confident on grating-present trials (blue) compared with grating-absent trials (orange). ***e***, Participants on average believed only ∼14% of trials to contain just noise (whereas the true proportion was 50%). ***f***, On grating-absent trials, participants reported seeing gratings with high confidence (3 of 4 or higher) on an average of 36% of trials. ***g***, There was a slightly higher, nonsignificant tendency to report orientations congruent with the expectation cue on grating-absent trials, which was driven by a few participants who were aware of the cue. ***h***, Participants' orientation response on the previous trial significantly predicted their orientation response on grating-absent trials; *** *p* < 0.001. Dots represent individual participants, and violin shapes indicate density. Error bars indicate within-subject SEM (***c***, ***d***) and SEM (***e***, ***f***, ***g***, ***h***).

Participants' accurately identified the grating orientation on grating-present trials more often than expected by chance (mean accuracy = 0.83, SD = 0.09; *t*_(24)_ = 18.1, *p* < 0.001). Furthermore, they were more accurate when they were confident they had seen a grating (i.e., higher than average confidence across trials) than when they were not (high, mean = 0.90, SD = 0.09; low, mean = 0.75, SD = 0.12; paired *t* test; *t*_(24)_ = 5.7, *p* < 0.001; Fig. 1*c*), demonstrating that they were able to perform the task and used the confidence ratings in a meaningful way. Participants also reported the perceived orientation more quickly on grating-present than grating-absent trials (*F*_(1,24)_ = 12.10, *p* = 0.002), as well as when they were more confident that they had seen a grating (*F*_(1,24)_ = 21.04, *p* < 0.001). Participants were more confident on grating-present trials (mean confidence = 2.49, SD = 0.60, on a scale of 1–4) than grating-absent trials (mean = 2.19, SD = 0.54; *t*_(24)_ = 4.76, *p* < 0.001; Fig. 1*d*). On debriefing, all participants but one underestimated the frequency of grating-absent trials, believing on average that 0.14 (SD = 0.13) of trials contained just noise, whereas the true proportion was 0.50 (Fig. 1*e*). Strikingly, participants reported perceiving a grating with high confidence (three of four or higher) on 36% of grating-absent trials (Fig. 1*f*). Surprisingly, the perceptual expectation cues did not significantly bias which orientation participants perceived on grating-absent trials (0.53 false percepts congruent with the cue, chance level is 0.50; *t*_(24)_ = 1.90, *p* = 0.07). The small numerical trend toward false percepts being congruent with the expectation cues was driven by a few individuals who became aware of the meaning of the cues (*N* = 7 of 25; Fig. 1*g*), potentially reflecting a concomitant response bias. High-confidence false percepts were not more affected by the cues than low confidence, that is, guessed, percepts (*t*_(23)_ = 0.37, *p* = 0.71). Trial-by-trial predictors of participants' choice behavior were explored using a logistic regression model (see above, Materials and Methods). As expected, orientation responses on grating-present trials were predominantly driven by the presented stimulus (*t*_(21)_ = 12.6, *p* < 0.001) but also by which orientation was perceived on the previous trial, such that the previously reported stimulus was more likely to be perceived again on the current trial (*t*_(21)_ = 5.3, *p* < 0.001). Interestingly, on grating-absent trials, previous percepts also significantly predicted orientation reports (*t*_(23)_ = 5.48, *p* < 0.001), whereas the cues did not (*t*_(23)_ = 2.03, *p* = 0.056; Fig. 1*h*). As above, the trend toward the cues influencing perception on grating-absent trials was driven by a few participants who became aware of the meaning of the cues. Thus, together, participants reported false percepts, but these were not significantly driven by the perceptual expectation cues in the present experiment, raising the possibility that these false percepts may have arisen from spontaneous fluctuations instead. We next investigated whether the false percepts were reflected in feedforward or feedback layers in the visual cortex.

**Figure 2.**
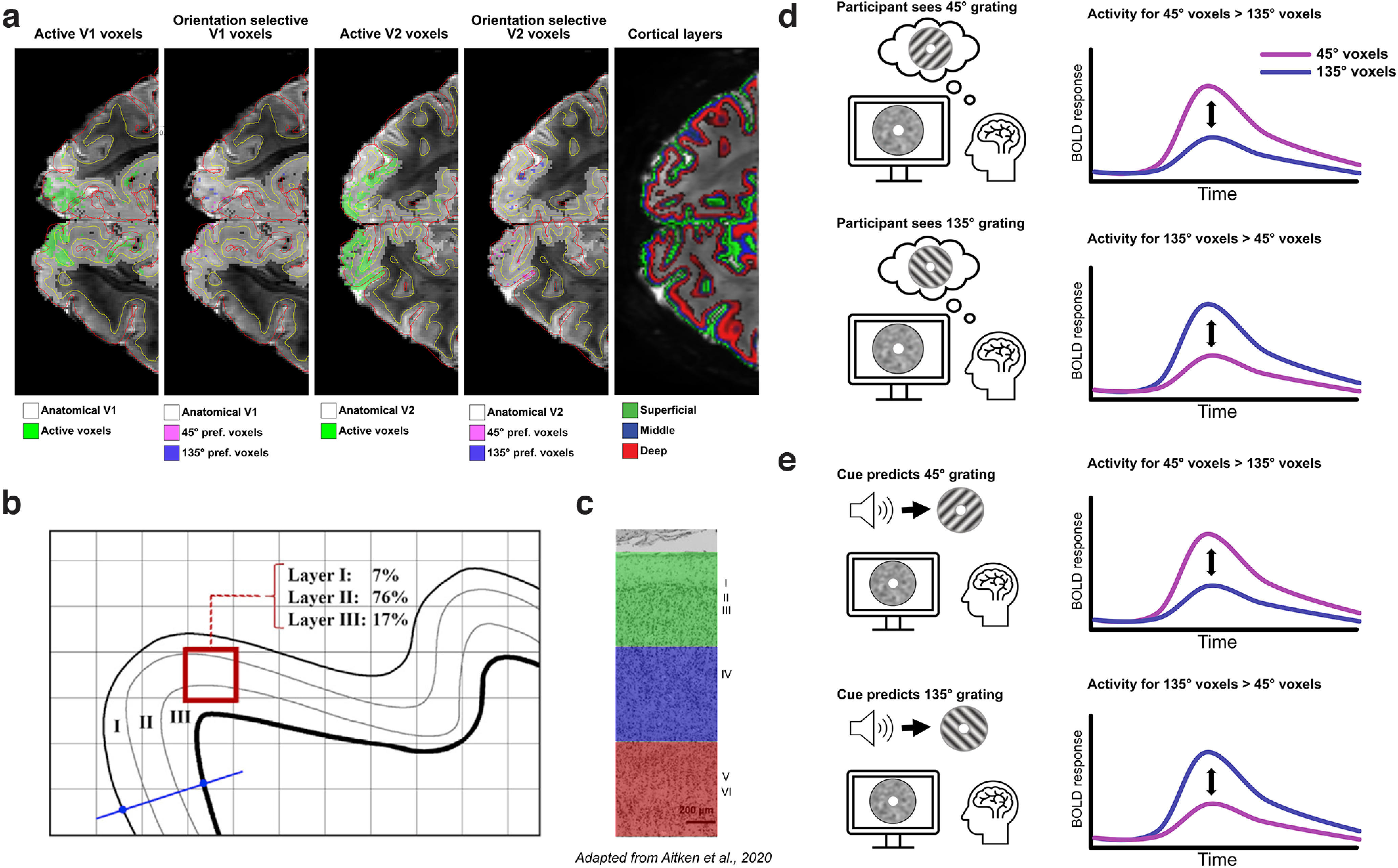
Schematic representation of the analysis pipeline. ***a***, Left, V1 and V2 voxels significantly activated by grating stimuli in a separate localizer run (green) were selected. Middle, Within this selection, the 500 voxels that mostly strongly preferred 45° over 135° gratings as well as the 500 voxels that mostly strongly preferred 135° over 45° were selected. Right, Gray matter was divided into superficial, middle, and deep layers. This figure is originally from Aitken et al., (2020b). ***b***, For each of the 500 voxels for each orientation, we calculated the contribution of each layer to that voxel. A spatial GLM was used to regress the contribution of each layer to the 500 voxels against the fMRI signal in these voxels, resulting in a single fMRI time course for superficial, middle, and deep layers for each set of orientation-preferring voxels. ***c***, The superficial (green), middle (blue), and deep (red) layers roughly correspond to layers I–III, IV, and V–VI respectively. Cytoarchitectural image of V1 adapted from de Sousa et al. (2010). ***d***, To calculate orientation-specific activity, BOLD activity was estimated for perceiving a 45° and a 135° grating in both 45°- and 135°-preferring voxels, separately for each layer. Activity in the incongruent ROI (e.g., perceiving 135°, 45°-preferring voxels) was subtracted from congruent ROI effects (e.g., perceiving 45°, 45°-preferring voxels), which were then averaged over the two orientation-preference ROIs. This resulted in layer- and orientation-specific activity for perceived (***d***) orientations. ***e***, The procedure was repeated for orientations predicted by the cue, resulting in layer- and orientation-specific activity for predicted orientations. Right, Time courses represent hypothesized BOLD responses to perceived and expected grating orientations in orientation-selective voxels.

**Figure 3.**
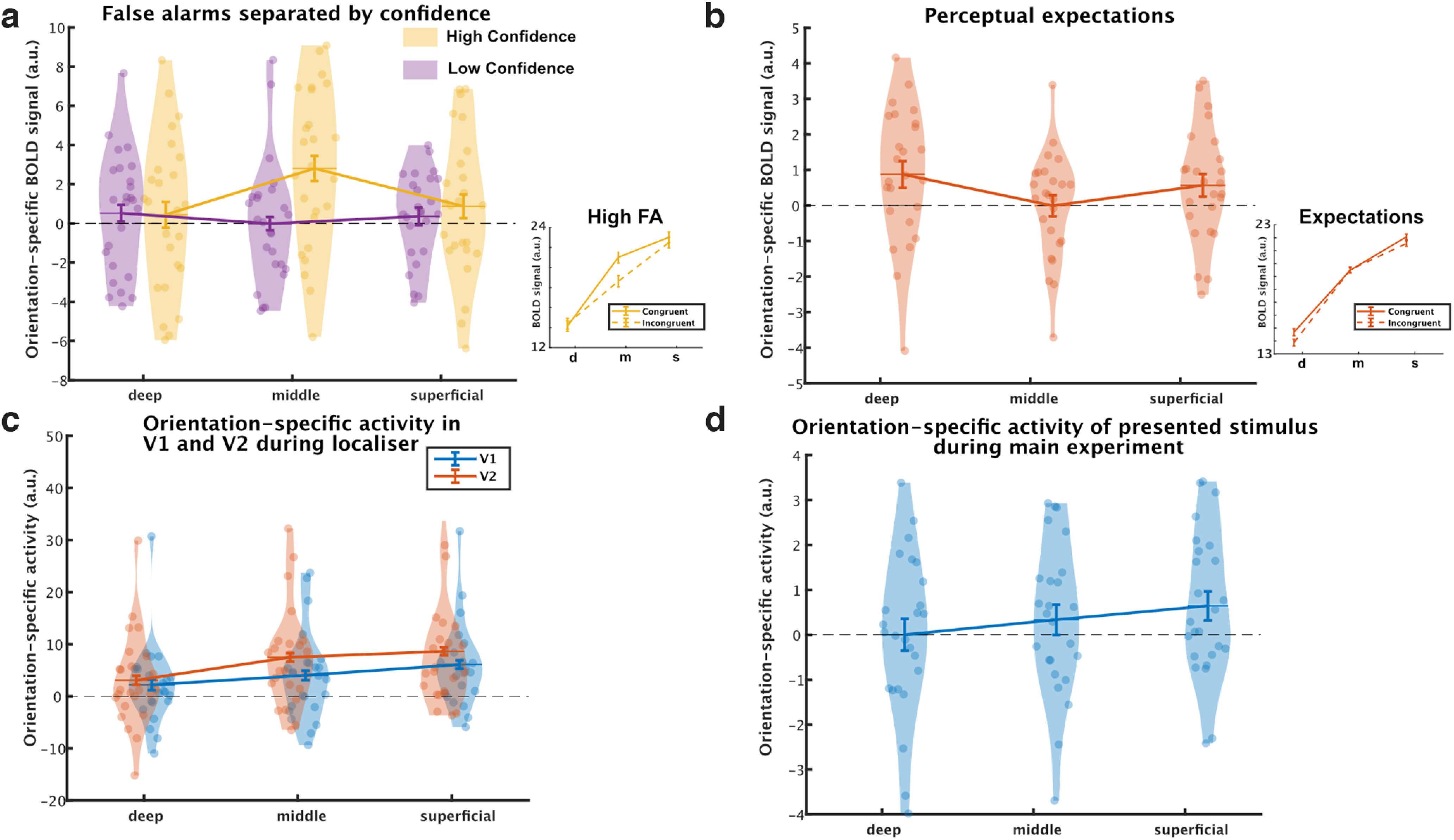
Orientation-specific BOLD activity in the cortical layers of V2. We calculated orientation-specific activity by separately estimating the effects of seeing or expecting 45° or 135° gratings in voxels that preferred 45° or 135° gratings on the basis of an independent localizer and subtracting incongruent activity (e.g., the effect of seeing 45° gratings in 135°-preferring voxels) from congruent activity (the effect of seeing 45° gratings in 45°-preferring voxels). Activity corresponding to perceived and expected gratings, respectively, were modeled separately on the same grating-absent trials. ***a***, The middle layers contained orientation-specific activity reflecting high-confidence false percepts (yellow), whereas low-confidence trials (purple) did not induce orientation-specific activity in any of the layers. ***b***, The deep and superficial layers reflected orientation-specific activity induced by perceptual expectations on grating-absent trials. ***c***, The localizer induced stronger stimulus-specific activity in V2 than in V1. ***d***, Stimulus-specific activity in the superficial layers evoked by presented gratings during the main experiment (grating-present trials). Error bars indicate within-subject SEM. FA = False Alarms. a.u. = arbitrary units.

### Orientation-specific activity reflecting false percepts and expectation cues in distinct cortical layers

In V2 the laminar profiles for false percepts with high confidence, false percepts with low confidence (guesses), and perceptual expectations were significantly different from each other (interaction between three conditions and three layers, *F*_(4,96)_ = 4.39, *p* = 0.003; see Fig. 3). High- and low-confidence false percepts were subsequently compared directly, revealing a significant difference in laminar profiles (interaction between two conditions and three layers, *F*_(2,48)_ = 5.42, *p* = 0.008). This was driven by increased orientation-specific activity for high- compared with low-confidence false percepts in the middle layers (paired *t* test, *t*_(24)_ = 3.26, *p* = 0.003), but not in the superficial (*t*_(24)_ = −0.73, *p* = 0.47) or deep (*t*_(24)_ = −0.10, *p* = 0.92) layers (see Fig. 3*a*). Thus, when participants reported perceiving a grating with a specific orientation with a high degree of confidence, although no such stimulus was present, there was activity specific to the reported grating orientation in the middle layers of V2 (one-sample *t* test, *t*_(24)_ = 3.43, *p* = 0.001). This high-confidence false percept related activity in the middle layers was significantly higher than in the deep (*t*_(24)_ = 2.51, *p* = 0.019) and superficial layers (*t*_(24)_ = 2.24, *p* = 0.034). No orientation-specific activity was present when the percept was reported with low confidence, that is, when the orientation report likely reflected a guess rather than a genuine perceptual experience (one-sample *t* test, *t*_(24)_ = −0.21, *p* = 0.51). A direct comparison of high-confidence false percepts and perceptual expectations demonstrated that they were associated with different laminar profiles (interaction between condition and layer *F*_(2,48)_ = 5.58, *p* = 0.007). This was primarily driven by a difference in middle layer activity (paired *t* test, *t*_(24)_ = 3.34, *p* = 0.003), which was activated by high-confidence false percepts (one-sample *t* test, *t*_(24)_ = 3.43, *p* = 0.001) but not by expectations (one-sample *t* test, *t*_(24)_ = −0.19, *p* = 0.51). Conversely, perceptual expectations evoked significant orientation-specific activity in the deep layers (see Fig. 3*b*; one-sample *t* test, *t*_(24)_ = 2.34, *p* = 0.014) and superficial layers (one-sample *t* test, *t*_(24)_ = 1.8, *p* = 0.042). Conversely, false percepts did not activate deep (one-sample *t* test, *t*_(24)_ = 0.56, *p* = 0.29) or superficial layers (one-sample *t* test, *t*_(24)_ = 1.19, *p* = 0.12), although the difference between false percepts and expectation-induced activity was not significant in the superficial and deep layers (all *p* values > 0.5). To investigate whether the results were driven by one of the two sets of orientation-preferring voxels, we repeated the repeated-measures ANOVAs with ROI as an added factor to explore whether our key effects interacted with the effect of ROI. Importantly, ROI did not interact with our effects of interest (all conditions * layer * ROI, *F*_(4,96)_ = 0.36, *p* = 0.84; high- versus low- confidence * layer * ROI, *F*_(2,48)_ = 0.0.28 *p* = 0.76). Thus, there was no evidence that the two orientation-specific ROIs responded differently under the conditions of interest.

In sum, high-confidence false percepts were reflected by activity specific to the perceived orientation in the middle layers, whereas perceptual expectations were related to activity specific to the cued orientation in the deep and superficial layers. Interestingly, the effect of perceptual expectations in the deep layers was not driven by those participants who became aware of the meaning of the cues (two-sample *t* test, *t*_(23)_ = 0.29, *p* = 0.78), and was significantly present in the subset of participants who were not aware of the meanings of the cues (*N* = 18, *t*_(17)_ = 2.20, *p* = 0.021). Together, these findings suggests that false percepts can arise from feedforward activity within a cortical circuit different from the one signaling perceptual expectations.

The effects reported above for V2 did not extend to V1 (all *p* values > 0.1). In fact, there was an interaction between layer (superficial, middle, and deep), stimulus condition (high- and low-confidence false percepts, and perceptual expectations), and ROI (V1 and V2; *F*_(4,96)_ = 3.42, *p* = 0.012), in line with the effects being specific to V2. This is likely explained by the relatively low spatial frequency (0.5 cycles/degree) gratings used here being more effective in activating V2 than V1, as has been demonstrated in animal study (Foster et al., 1985). In line with this, a cross-validated analysis of orientation-specific BOLD signals within the functional localizer revealed stronger orientation-specific effects in V2 than V1 across all layers (main effect of ROI, *F*_(1,23)_ = 23.56, *p* < 0.001; all layers, *p* < 0.01; see Fig. 3*c*). However, the degree to which we can make separate inferences about V1 and V2 are limited. The anatomic ROIs share overlaps because they represent probability maps, which share voxels at the border as these voxels could belong to either V1 or V2. Exploring the effects of the presented orientation on grating-present trials, we find that these low-contrast gratings embedded in noise evoked significant orientation-specific activity in the superficial layers of V2 (*t*_(24)_ = 2.00, *p* = 0.028) but not the other layers (both *p* values > 0.1; see Fig. 3*d*). It is perhaps not surprising that such weak and noisy stimuli did not evoke a significant orientation-specific BOLD signal in the deep and middle layers, but it is striking to note that more cognitive processes like expectation and perception did.

### Noise patch control analyses

Given that the false percepts on noise-only trials were reflected in the middle input layers of V2, one might be concerned that the noise patches themselves contained orientation signals that drove our effects. To avoid this issue a priori, we generated four noise patches with a flat orientation energy spectrum, as determined by a bank of orientation filters based on Wyart et al. (2012; see above, Materials and Methods). However, participants were still significantly biased toward specific orientations for some of the noise patches. Specifically, noise patches 2 and 3 were significantly more often identified as 45° than 135° (63 and 69%, respectively; *t*_(24)_ = 2.82, *p* = 0.01 and *t*_(24)_ = 5.3, *p* < 0.01), and vice versa for noise patch 4 (71%, *t*_(24)_ = 5.77, *p* < 0.01). Noise patch 1 showed no significant bias (54% identified as 135°, *t*_(24)_ = 1.31, *p* > 0.1). In theory, the stimulus-specific activity reported in the middle layers of V2 on the high-confidence false alarm trials could therefore be driven by an unspecified signal present in the noise patches themselves. To investigate this possibility, we performed two control analyses.

First, if the noise patches were driving the results, the effects should disappear if all conditions of interest (i.e., low- and high-confidence false percepts of either orientation) were modeled separately for each noise patch and then averaged over noise patches afterward to ensure all patches contribute to both 45° and 135° percepts equally. In other words, in such an analysis each noise patch contributes equally to the estimated BOLD activity for 45° percepts and 135° percepts, regardless of participants' propensity to perceive them as one more often than the other. In fact, this analysis replicated our core finding, namely, that high-confidence false percepts were reflected by increased middle layer activity compared with low-confidence false percepts (*t*_(24)_ = 2.51, *p* = 0.019). Indeed, there was significant orientation-specific activity in the middle layers for high-confidence false percepts (*t*_(24)_ = 3.09, *p* = 0.003), but not in the other layers (*p* > 0.1). Therefore, when eliminating the possible confounding effect of the noise patches, our effects remain.

Second, we estimated any potential orientation-specific effects evoked by the different noise patches directly, comparing them against each other to explore any differences in stimulus-specific activity. We did not find any evidence that the noise patches alone caused stimulus-specific activity (across all combinations and layers, *p* > 0.05). Together, the results of these control analyses are not in line with an explanation of our effects in terms of stimulus-driven signals in the noise patches themselves but instead strongly suggest that they reflect internally generated percepts.

### High-confidence false percepts and reduced sensory precision predict everyday hallucination severity

In a separate online experiment (*N* = 100), we tested whether the high-confidence false percepts that were related to middle layer activity in the layer-specific fMRI study correlated with the prevalence and severity of hallucinatory percepts in daily life as measured by the CAPS questionnaire (Bell et al., 2006). The false percept task used here was similar to the one used in the fMRI experiment (with slight variations in practice procedure and trial counts; see above, Materials and Methods). One important difference was the introduction of three (rather than one) contrast levels on the grating-present trials to enable estimates of sensory precision, that is, how task accuracy depended on evidence quality. Specifically, a base-level contrast value was selected for each participant based on their performance during the practice phase, and this base contrast was used during the main experiment along with gratings with 1% higher and 1% lower contrast.

In the online study, 22 of the 100 participants became aware of the meaning of the cue as revealed by the debriefing questionnaire. Confidence in having seen an increase with grating contrast (0%, base − 1%; base, base + 1%; *F*_(2,297)_ = 133.8, *p* < 0.001; all *post hoc* tests, *p* < 0.001), but there was no effect of cue validity on confidence (*F*_(1,99)_ = 1.952, *p* = 0.17), nor was there an interaction between cue validity and stimulus contrast (*F*_(1,99)_ = 0.87, *p* = 0.42). Accuracy increased with contrast (*F*_(2,196)_ = 49.3, *p* < 0.001) and was lower when the expectation cue was invalid (*F*_(1,98)_ = 20.50, *p* < 0.001). Participants who became aware of the meaning of the cue showed stronger cues effects on accuracy as shown by a group by cue effect interaction (*F*_(1,98)_ = 10.7, *p* = 0.001). Furthermore, the expectation cues influenced participants' choice behavior on grating-absent trials (*t*_(99)_ = 2.97, *p* = 0.004). This was driven by participants who became aware of the cue meaning (*N* = 22, 58.3% false percepts congruent with the cue), who were significantly more influenced by the cues than those who were not aware of their meaning (*N* = 78, 51.6% false percepts congruent with the cue; *t*_(98)_ = 2.83, *p* = 0.006). Those unaware of the meaning of the cues only showed trend-level responses in line with the cue (*t*_(77)_ = 1.87, *p* = 0.065), whereas those aware showed a significant effect of cue (*t*_(21)_ = 2.41, *p* = 0.025). These findings are similar to those of the fMRI study, where the effect of cue was also driven by those aware of the purpose of the cues. Further, accuracy and confidence increased with grating contrast as expected. We modeled choice behavior on grating-absent and grating-present trials using a logistic regression model. This revealed that responses on grating-present trials were driven by the interaction of the current stimulus and contrast (hereafter referred to as sensory precision; *t*_(99)_ = 14.11, *p* < 0.001), previous response (*t*_(99)_ = 8.93, *p* < 0.001), and current stimulus (*t*_(99)_ = 8.07, *p* < 0.001). Conversely, responses on grating-absent trials were driven by previous responses (*t*_(99)_ = 2.34, *p* = 0.021).

**Figure 4.**
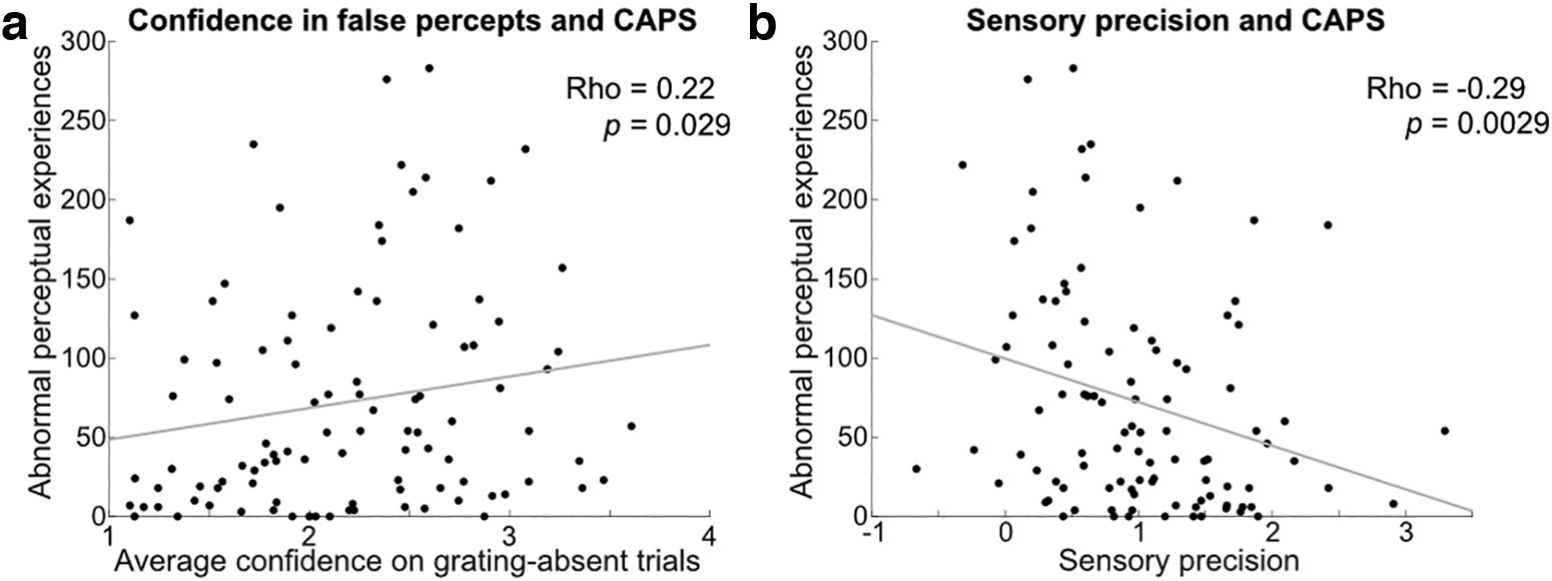
Correlations between grating percepts in the current task and everyday hallucinations. ***a***, Abnormal perceptual experiences correlated with confidence in false percepts. ***b***, Abnormal perceptual experiences correlated with less reliance on sensory precision (i.e., the interactive effect of grating contrast and orientation on choice behavior). CAPS = Cardiff Anomalous Perceptions Scale score.

Crucially, the prevalence of abnormal perceptual experiences in daily life (total CAPS scores; Bell et al., 2006) was positively correlated with the average confidence that participants reported on grating-absent trials, that is, the prevalence of high-confidence false percepts in our task, across participants in the online sample (Rho = 0.22, *p* = 0.029; Fig. 4*a*). Further, the sensory precision term, the influence of grating contrast on choice behavior, correlated negatively with abnormal perceptual experience scores (Rho = −0.30, *p* = 0.003; Fig. 4*b*). In other words, the less sensitive participants were to stimulus contrast, the more likely they were to experience abnormal perceptual experiences in daily life. Using a linear regression model, both confidence on grating-absent trials (*t*_(99)_ = 2.01, *p* = 0.048) and sensory precision (*t*_(99)_ = −2.98, *p* = 0.004) were found to be separate predictors of abnormal perceptual experience severity (overall linear regression model, *F*_(2,97)_ = 6.12, *p* = 0.003, *R*^2^ = 0.112). We did not find a relation between average confidence on grating-absent trials and delusion ideation (Rho = 0.13, *p* = 0.20), but there was a correlation with the sensory precision term (Rho = −0.29, *p* = 0.004). In sum, high-confidence false percepts of oriented gratings were related to the severity of everyday abnormal perceptual experiences. Given that the fMRI study showed that such high-confidence false percepts were reflected by stimulus-like signals in the middle input layers of the early visual cortex, this raises the possibility that abnormal perception in everyday life may partly result from similar stimulus-like sensory fluctuations.

## Discussion

There is a wide range of theories that attempt to explain the neural mechanisms of false percepts. The dominant theory highlights the role of feedback prediction signals in driving false percepts (Corlett et al., 2019; Powers et al., 2016; Reichert et al., 2013; Sterzer et al., 2018). Others have proposed that false percepts can emerge in feedforward fashion as well (Burke, 2002; Hahamy et al., 2021). Here, we tested feedforward and feedback contributions to false percepts using layer-specific fMRI. We found that the deep layers of the visual cortex reflected perceptual expectations, replicating previous research (Aitken et al., 2020b). However, high-confidence false percepts were reflected solely in the middle input layers of the early visual cortex and were unaffected by perceptual expectation cues. These high-confidence false percepts correlated with everyday hallucination severity in a separate online study. This suggests that false percepts can arise from stimulus-like feedforward activity in sensory cortex and do not necessarily require feedback-induced stimulus templates in the deep layers of the visual cortex.

The core finding here—that orientation-specific activity in the middle layers of V2 can lead participants to perceive a grating that was not actually presented—has important implications for the field of hallucination research as well as perception research more generally. That is, it suggests that false percepts can arise through activity in the input layers in the absence of top-down stimulus templates induced by perceptual expectations. This puts a larger emphasis on feedforward signals than previously assumed in dominant models of hallucinations, which have largely focused on the role of top-down expectations in driving false percepts (Corlett et al., 2019; Haarsma et al., 2022; Kafadar et al., 2020; Powers et al., 2016, 2017; Schmack et al., 2021; Sterzer et al., 2018). Thus, this research adds to the diversity of mechanisms that might underlie false inferences.

These findings are in line with theories that state that false percepts can arise from spontaneous activity that resembles sensory input (Burke, 2002), which recent studies have confirmed in neurologic disorders like Charles Bonnet Syndrome (Hahamy et al., 2021). Furthermore, they expand on previous studies that have found that early sensory activity can lead to false alarms (Boly et al., 2007; Busch et al., 2009; Pajani et al., 2015; Podvalny et al., 2019; Ress and Heeger, 2003; Wyart and Tallon-Baudry, 2009) by suggesting that the activity reported by these studies may reflect spontaneous fluctuations in the input layers rather than feedback from higher-order regions. Recent circular inference models of hallucinations have emphasized the role of ascending loops, akin to feedforward activity, in unimodal hallucinations as seen in psychotic disorders (Denève and Jardri, 2016; Leptourgos et al., 2022). Specifically, they suggest that weak sensory signals can trigger perceptual hypotheses that are then counted as sensory evidence themselves in runaway overcounting loops. This overcounting of sensory signals has been shown to correlate with positive symptoms (e.g., hallucinations and delusions) in schizophrenia patients (Jardri et al., 2017).

Top-down processes like visual working memory and perceptual expectations induce stimulus representations in the deep and superficial but not the middle layers of the early visual cortex (Aitken et al., 2020b; Lawrence et al., 2018; van Kerkoerle et al., 2017). However, keeping an image in visual working memory or merely expecting a visual stimulus does not lead to a concurrent perceptual experience, as is the case with a hallucination. One interpretation therefore might be that the middle layers are required to be activated in order for a perceptual experience to occur that is attributed externally. Indeed, it has been suggested that feedforward signals are essential in distinguishing imagination from veridical perception (Dijkstra et al., 2022).

The prevalence of high-confidence false percepts in our experiment correlated with everyday abnormal perceptual experiences. This is in line with previous studies demonstrating that those who hallucinate are more prone to perceive stimuli in noise in detection tasks (Haarsma et al., 2020; Kafadar et al., 2020; Powers et al., 2017; Stuke et al., 2021). Furthermore, higher hallucination scores correlated negatively with the sensory precision term of our logistic regression model. That is, the less participants relied on the contrast of the sensory stimulus in making their perceptual decision, the more they experienced hallucinations in everyday life. This is in line with Bayesian models of hallucinations, where a reduction in sensory precision increases the influence of prior expectations, possibly leading to hallucinations (Adams et al., 2013). Interestingly, reduced reliance on sensory contrast on the one hand, and confidence on grating-absent trials on the other, were separate predictors of everyday hallucinations severity, suggesting separate underlying mechanisms contributing to hallucinations.

These findings should not be taken as evidence against the theory that top-down perceptual expectations can play an important part in generating hallucinations, for which there is ample indirect evidence (Cassidy et al., 2018; Haarsma et al., 2020; Kafadar et al., 2020; Powers et al., 2017; Schmack et al., 2021; Stuke et al., 2021; Teufel et al., 2015; Zarkali et al., 2019). Instead, these findings highlight that the possibility that false percepts could occur in principle from feedforward activity in early sensory regions, potentially mapping onto different forms of hallucinations such as so-called minor phenomena and complex visual hallucinations, respectively (Mocellin et al., 2006; Pagonabarraga et al., 2016). Unlike the false percepts studied here, hallucinations induced by perceptual expectations might be reflected by signals in the agranular layers, mimicking the expectation effects in the present study. An interesting question is whether agranular feedback signals on their own are sufficient to generate a perceptual experience, or whether additional activity in the input layers is required. Speculatively, to experience expectation-induced hallucinations, feedback signals may need to override activity in the middle input layers. Further, top-down expectation effects might still play a role in the present study as there was a strong expectation of stimulus presence, regardless of content. That is, participants strongly expected to see a grating on every trial, even if they were not sure which orientation it would have. Interestingly, expectations about stimulus presence versus absence and expectations about stimulus content have been suggested to be supported by different neural processes (Mazor et al., 2020; Podvalny et al., 2019; Samaha et al., 2020). Future research could elucidate the contribution of stimulus presence expectations on the effects found in the present study by manipulating this explicitly.

Finally, in theory, the V2 middle layer signals reflecting false percepts in the current study could have resulted from feedback signals to V1 or the thalamus being sent downstream to the middle layers of V2. Such an indirect feedback effect does not seem in line with the absence of stimulus-specific effects reflecting false percepts in V1 or the lack of an effect of the cued orientations on behavior. However, it should be acknowledged that indirect feedback effects cannot be fully ruled out on the basis of a null result.

Expectations did not affect perception in the present study. We speculate this might be because of the normative sample, as well as the expectations being implicit. Indeed, there is increasing evidence that conscious expectations exert stronger effects on perception than unconscious expectations (Alilović et al., 2021; Meijs et al., 2018). Studies that do report effects of implicit cues on perception typically reveal biased perception of existing stimuli rather than eliciting percepts *de novo* (Aitken et al., 2020a; Kok et al., 2013); although Chalk et al. (2010) have a notable exception.

Despite the expectation cues not affecting perception, they did induce orientation-specific templates in the deep layers of the early visual cortex, in line with previous work (Aitken et al., 2020b). Interestingly, in contrast to the previous study, there was also significant expectation-evoked activity in the superficial layers. This additional effect might be explained because of the concurrent presentation of noisy stimuli in this study, whereas no stimulus was presented in the previous study. That is, speculatively, the presence of (noisy) sensory input may unlock modulatory effects of feedback in the superficial layers. Strikingly, the representation of expected orientations in the deep layers was reliable even in those who were not aware of the cue–stimulus relationship, which suggests that the brain can generate sensory expectations based on statistical relationships that are learned outside of conscious awareness (Aitken and Kok, 2022).

In the present study, no reliable effects of either perceptual expectations or false percepts were found in V1. This could be because of the lower spatial frequency stimuli used in the present study (0.5 cycles/degree) compared with previous studies that reported orientation-specific effects in V1 (1.0–1.5 cycles/degree; Kok et al., 2014; Lawrence et al., 2018, 2019a) as V2 neurons prefer lower spatial frequencies than V1 neurons (Foster et al., 1985). This was confirmed by our localizer analyses, showing stronger orientation-specific effects in V2 than in V1. However, we do not make strong claims about our effects being specific to V2, given that V1 neurons do respond to low frequencies as well, particularly in the periphery (Broderick et al., 2022). Furthermore, the V1 and V2 ROIs used here were generated by transforming surface-based labels to volume space, leading to some overlap between the two ROIs, particularly near the foveal confluence, limiting the degree to which we can make inferences about whether a particular effect pertains to V1 or V2.

Here, we investigated stimulus-specific effects using oriented gratings, which likely result from inhomogeneities in the spatial distribution of orientation preferences across the visual field, such as the well-known radial bias (Freeman et al., 2013; Roth et al., 2022). Whether the present results also hold for other stimulus types, such as abstract shapes or complex objects, is an important question for future research.

In conclusion, high-confidence false percepts were reflected by orientation-specific activity in the middle input layers of the early visual cortex, whereas perceptual expectations activated the deep layers. These findings suggest that false percepts can arise from low-level content-specific fluctuations in the input layers of the visual cortex. This nuances the view that false percepts are necessarily driven by top-down expectations (Corlett et al., 2019; Powers et al., 2016; Sterzer et al., 2018). Future studies should aim to further explore the nature of these low-level fluctuations and what drives them, as well as investigate whether false percepts can also be driven by purely top-down signals. These findings have important implications for our understanding of the neural mechanisms underlying hallucinations, revealing how the brain can generate perception in the absence of sensory input.
